# Farnesysltransferase Inhibitor Prevents Burn Injury-Induced Metabolome Changes in Muscle

**DOI:** 10.3390/metabo12090800

**Published:** 2022-08-27

**Authors:** Harumasa Nakazawa, Lai Ping Wong, Laura Shelton, Ruslan Sadreyev, Masao Kaneki

**Affiliations:** 1 Department of Anesthesia, Critical Care and Pain Medicine, Massachusetts General Hospital, Harvard Medical School, 149 Thirteenth Street, Charlestown, MA 02129, USA; 2 Shriners Hospitals for Children, 51 Blossom Steet, Boston, MA 02114, USA; 3 Department of Molecular Biology, Massachusetts General Hospital, Harvard Medical School, 55 Fruit Street, Boston, MA 02114, USA; 4 Human Metabolome Technologies, 24 Denby Rd., Boston, MA 02134, USA

**Keywords:** burn injury, metabolomics, skeletal muscle, farnesyltransferase inhibitor

## Abstract

Burn injury remains a significant public health issue worldwide. Metabolic derangements are a major complication of burn injury and negatively affect the clinical outcomes of severely burned patients. These metabolic aberrations include muscle wasting, hypermetabolism, hyperglycemia, hyperlactatemia, insulin resistance, and mitochondrial dysfunction. However, little is known about the impact of burn injury on the metabolome profile in skeletal muscle. We have previously shown that farnesyltransferase inhibitor (FTI) reverses burn injury-induced insulin resistance, mitochondrial dysfunction, and the Warburg effect in mouse skeletal muscle. To evaluate metabolome composition, targeted quantitative analysis was performed using capillary electrophoresis mass spectrometry in mouse skeletal muscle. Principal component analysis (PCA), partial least squares discriminant analysis (PLS-DA), and hierarchical cluster analysis demonstrated that burn injury induced a global change in metabolome composition. FTI treatment almost completely prevented burn injury-induced alterations in metabolite levels. Pathway analysis revealed that the pathways most affected by burn injury were purine, glutathione, β-alanine, glycine, serine, and threonine metabolism. Burn injury induced a suppressed oxidized to reduced nicotinamide adenine dinucleotide (NAD^+^/NADH) ratio as well as oxidative stress and adenosine triphosphate (ATP) depletion, all of which were reversed by FTI. Moreover, our data raise the possibility that burn injury may lead to increased glutaminolysis and reductive carboxylation in mouse skeletal muscle.

## 1. Introduction

Severe burn injury remains a significant public health issue associated with high morbidity and mortality rates and therefore represents a major challenge in critical care [1]. In 2004, nearly 11 million people worldwide were severely burned resulting in an estimated 180,000 deaths annually (WHO, Burns fact sheet. 2018).

Metabolic derangements are a major complication of burn injury and negatively affect clinical outcomes of severely burned patients [2,3]. These metabolic alterations include muscle wasting, hypermetabolism, insulin resistance, hyperglycemia, hyperlactatemia, and mitochondrial dysfunction [3,4,5,6,7,8,9]. Muscle wasting worsens clinical outcomes in burn patients. It makes weaning off from mechanical ventilation difficult, which increases the risk of secondary pulmonary infection and lung injury, leading to prolonged hospital stays and increased mortality in severely burned patients [10]. Moreover, muscle wasting causes muscle weakness-related decreased activities of daily living (ADL), resulting in prolonged rehabilitation and decreased quality of life (QOL) for those surviving the acute phase of burn injury [11]. Muscle cachexic changes have been implicated in the pathogenesis of burn injury [8,12], although direct evidence is lacking.

Hyperlactatemia and lactic acidosis are predictors for worse prognosis and mortality in burn patients [5,6,13,14,15,16]. Previous studies have shown that over and above the effects of hypoperfusion and tissue hypoxia, metabolic alterations (i.e., the Warburg effect) contribute to hyperlactatemia in burn patients [13] and patients with shock [17]. The Warburg effect (aka aerobic glycolysis) is a metabolic shift in which glycolysis-mediated ATP synthesis predominates over that of oxidative phosphorylation in the mitochondria even in the presence of sufficient oxygen availability, which leads to increased production and secretion of lactic acid. Hypoxia-inducible factor (HIF)-1α is a transcription factor that orchestrates the Warburg effect. Skeletal muscle is a major source of lactate in circulation.

Burn injury induces insulin resistance in skeletal muscle [18,19,20], which, in turn, contributes to hyperglycemia and muscle wasting in severely burned patients, since skeletal muscle is the largest organ that takes up glucose from the circulation, and insulin action plays a key role in the maintenance of muscle mass [21,22]. Inflammatory/stress response and inducible nitric synthase (iNOS) play an important role in burn injury-induced insulin resistance [9,18,19,23].

Mitochondrial dysfunction/disintegrity is another feature of burn injury-induced metabolic aberrations [23,24,25]. Burn injury-induced mitochondrial dysfunction/disintegrity includes decreased oxygen consumption rate by the electron transport chain (e.g., complex I), morphological alterations, and loss of mitochondrial DNA in skeletal muscle [23,26,27]. However, little is known about changes in metabolites that are associated with insulin resistance and mitochondrial dysfunction in burns.

We have previously shown in mice [20,23] that: (1) burn injury induces insulin resistance, the Warburg effect, and mitochondrial dysfunction/disintegrity in mouse skeletal muscle at 3 days after burn injury compared with a sham burn; and (2) farnesyltransferase inhibitor, FTI-277, reverses the burn injury-induced metabolic alterations. The burn injury-induced Warburg effect in skeletal muscle was indicated by markedly increased expression of HIF-1α and its downstream glycolytic genes, Glut1, lactate dehydrogenase A (LDHA), pyruvate dehydrogenase (PDH) kinase 1 (PDK1) and pyruvate kinase M2, and increased ex vivo lactate secretion by skeletal muscle in mice [23]. However, limited knowledge is available about the effects of burn injury on the metabolome profile in skeletal muscle. Therefore, we studied the effects of burn injury and FTI-277 on levels of metabolites by metabolomics in mouse skeletal muscle.

## 2. Results and Discussion

### 2.1. Multivariate Analysis

**Figure 1:**
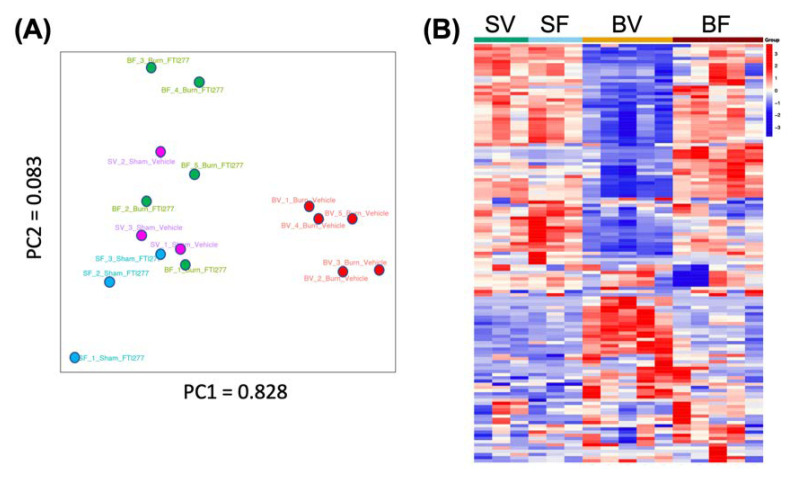
PCA score scatter plot (**A**) and the heat map (**B**). (**A**) Principal component scores are plotted. Purple: Sham-Vehicle, Blue: Sham-FTI, Red: Burn-Vehicle, Green: Burn-FTI. (**B**) SV: Sham-Vehicle, SF: Sham-FTI, BV: Burn-Vehicle, BF: Burn-FTI.

Principal component analysis (PCA) demonstrated a clear clustering of the Burn-Vehicle group that was separate from the other three groups, namely Sham-Vehicle, Sham-FTI, and Burn-FTI (Figure 1A). The first principal component (PC1) accounted for 82.8% of the variability and separated Burn+Vehicle from the other three groups. PC2 accounted for 8.3% of the variability. Sham-Vehicle, Sham-FTI, and Burn+FTI were relatively similar to each other. For the first principal component (PC1), we searched for the top ten metabolites based on the absolute values of the positive and negative factor loadings. The top ten metabolites with the highest PC1 factor loadings are listed in Table 1. All the 10 metabolites with the highest absolute factor loadings were negative factors and significantly lower in Burn-Vehicle compared with the other three groups (Figure S1). On the other hand, the loading values of the top five metabolites with the highest positive factor loadings were less than 0.02 (Table S1), while those of the top five negative factor loadings were greater than 0.06 (Table 1). Although there were some metabolites that were increased in Burn-Vehicle compared with the other three groups as shown below, one of the characteristics of the metabolome profile of Burn-Vehicle could be deficiency of some metabolites as those listed in Table 1 (Figure S1). Analysis of the overall metabolome profiles suggested that burn injury-induced substantial changes in levels of many metabolites in Burn-Vehicle, leading to a clearly distinct profile as compared to the other three groups as shown in the heatmap. (Figure 1B).

**Figure 2:**
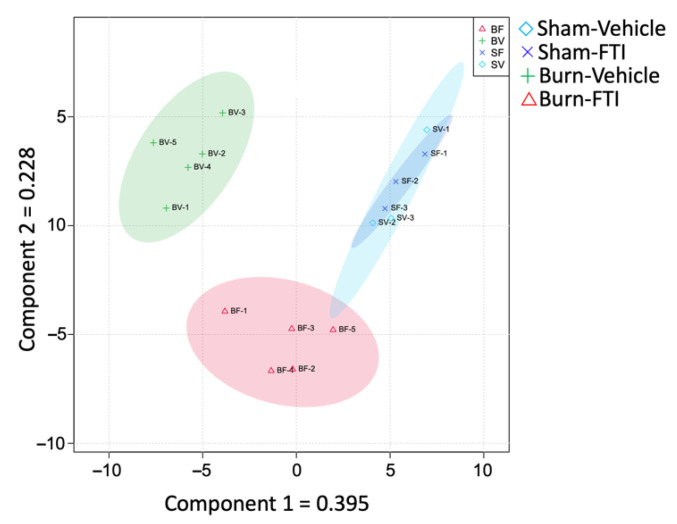
PLS-DA score scatter plot. Blue diamond: Sham-Vehicle, Blue cross mark: Sham-FTI, Green plus mark: Burn-Vehicle, Red triangle: Burn-FTI.

To further investigate the separation between the four groups based on combined levels of all metabolites, we used partial least square discrimination analysis (PLS-DA) [28]. The resulting PLS-DA score scatter plot of the four groups (Figure 2) suggests that Sham-Vehicle, Burn-Vehicle and, Burn-FTI are well separated from each other, whereas the Sham-FTI samples are grouped together with the Sham-Vehicle samples.

**Figure 3:**
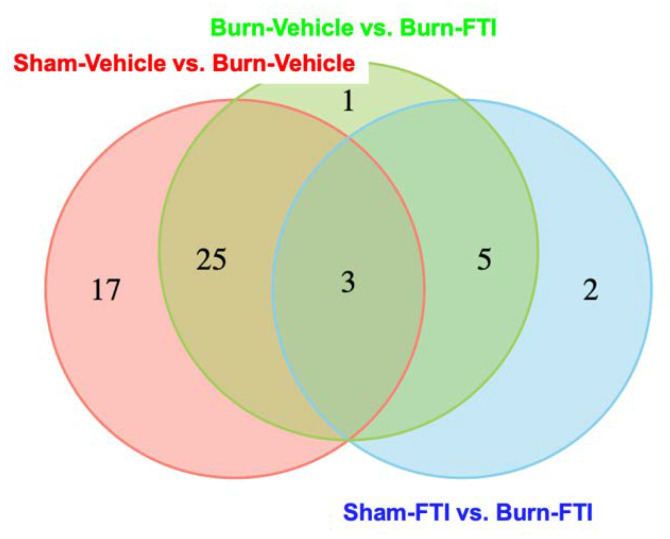
The intersection of differential metabolites. Venn diagram illustrates the distribution of differential metabolites showing significantly (*p* < 0.05 by Wilcoxon test) modulated levels. Red, blue and green circles indicate differential metabolites between Sham-Vehicle vs. Burn-Vehicle, those between Sham-FTI vs. Burn-FTI, and those between Burn-Vehicle vs. Burn-FTI, respectively.

Next, we identified differential metabolites between Sham-Vehicle and Burn-Vehicle, between Sham-FTI and Burn-FTI, and between Burn-Vehicle and Burn-FTI. Differential metabolites were defined as those with an absolute fold change > 2 and *p*-value < 0.05. The numbers of differential metabolites unique to and shared across the three comparisons are shown in Figure 3 and Figure S2. For differential metabolite analysis, we used both the Wilcoxon rank sum test (Figure 3) and the Student *t*-test (Figure S2). The results produced by these two tests were largely consistent. There are significant numbers of differential metabolites between Sham-Vehicle and Burn-Vehicle (*n* = 45) and between Burn-Vehicle and Burn-FTI (*n* = 34) (Figure 3). On the other hand, there were a relatively small number of differential metabolites (*n* = 10) between Sham-FTI and Burn-FTI (Figure 3). Among the differential metabolites between Sham-Vehicle and Burn-Vehicle, 34 metabolites were significantly lower in Burn-Vehicle compared with Sham-Vehicle, while 11 metabolites were significantly higher in Burn-Vehicle. Consistent with the result showing that all the top ten metabolites with the highest absolute PC1 factor loadings were negative factors, these data support the notion that a characteristic of burn injury-induced metabolic dysfunction may be suppressed abundance of certain metabolites. On the other hand, no differential metabolites were found between Sham-Vehicle and Sham-FTI.

### 2.2. Effects of Burn Injury—Volcano Plot and Pathway Analysis

**Figure 4:**
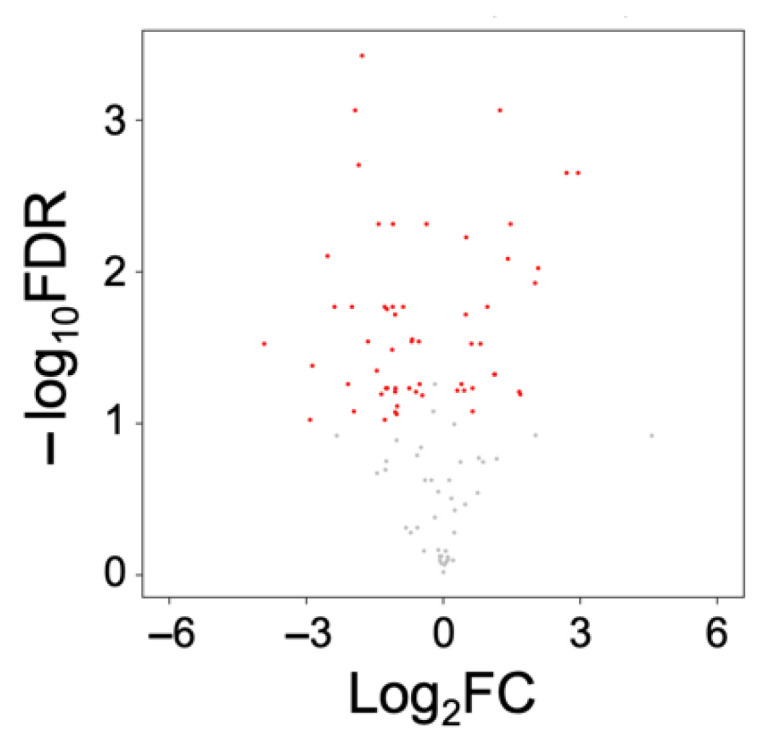
Volcano plot of altered metabolites between Sham-Vehicle and Burn-Vehicle. Metabolites with an absolute fold change (FC) > 1.2 and false discovery rate (FDR) < 0.10 are plotted. Red: FDR < 0.1, Grey: ≥ 0.1.

The multivariate analyses showed that the metabolome profile of Burn-Vehicle is clearly segregated from those of the other three groups. Therefore, we analyzed the effects of burn injury mainly comparing Sham-Vehicle with Burn-Vehicle. Since comparison between Sham-FTI and Burn-Vehicle has little biological relevance, we did not compare between Sham-FTI and Burn-Vehicle. Consistent with the results of PCA and PLS-DA and the number of differential metabolites, the volcano plot between Sham-Vehicle and Burn-Vehicle shows that a number of metabolites were significantly altered by burn injury and that more metabolites were negatively modulated (fold change < 0) by burn injury compared with positively modulated metabolites (Figure 4).

Pathway analysis identified metabolic pathways that were significantly changed between Burn + Vehicle and Sham + Vehicle (*p* < 0.05) (Table 2). The most affected pathways (FDR < 0.05, impact > 0.1) are purine, glutathione, β-alanine, glycine, serine, and threonine metabolism.

**Figure 5:**
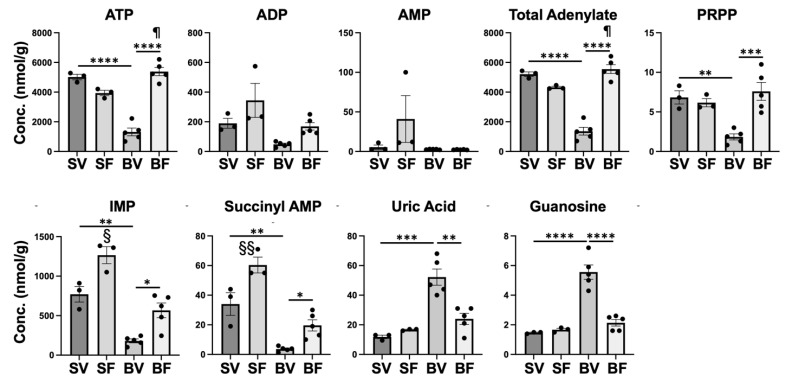
Effects of burn injury and FTI-277 on metabolites related to purine metabolism. SV: Sham-Vehicle; SF: Sham-FTI; BV: Burn-Vehicle; BF: Burn-FTI, Succinyl AMP: adenylosuccinic acid, * *p* < 0.05, ** *p* < 0.01, *** *p* < 0.001, **** *p* < 0.0001, § *p* < 0.05 vs. Sham-Vehicle, *p* < 0.001 vs. Burn-FTI, §§ *p* < 0.01 vs. Sham-Vehicle, *p* < 0.001 vs. Burn-FTI, ¶ *p* < 0.05 vs. Sham-FTI.

Related to purine metabolism (Figure S3), ATP was markedly lower in Burn-Vehicle compared with Sham-Vehicle and Burn-FTI (Figure 5) consistent with our previous study [26]. There were no significant differences in ADP and AMP between Sham-Vehicle and Burn-Vehicle, although ADP appeared to be lower in Burn-Vehicle compared with Sham-Vehicle (*p* > 0.10). As a result, total adenylate (ATP + ADP + AMP) was markedly lower in Burn-Vehicle compared with Sham-Vehicle and Burn-FTI. GTP tended to be lower in Burn-Vehicle compared with Sham-Vehicle, although there was no significant difference (*p* < 0.10) (Figure S4). There was no significant difference in GDP between Burn-Vehicle and Sham-Vehicle. On the other hand, GMP was significantly higher in Burn-Vehicle compared with Sham-Vehicle and Burn-FTI (Figure S4).

Phosphoribosylpyrophosphate (PRPP) and glutamine are converted to inosine 5′-monophosphate (IMP) by the de novo purine synthesis pathway and then converted to adenylosuccinic acid (succinyl AMP) or xanthine monophosphate (XMP) (Figure S3). The salvage pathways of purine synthesis also use PRPP as a substrate. PRPP, IMP and adenylosuccinic acid (succinyl AMP) were significantly lower in Burn-Vehicle compared with Sham-Vehicle and Burn-FTI (Figure 5). Adenine was undetectable in three of five mice in Burn-Vehicle, although adenine was detectable in all the mice of the other three groups. Glutamine was significantly lower in Burn-Vehicle compared with Sham-Vehicle and Burn-FTI (Figure S1). On the other hand, there was no significant difference in XMP between the four groups, although XMP appeared to be higher in Burn-Vehicle (*p* > 0.10) (Figure S4). These data suggest that purine synthesis may not be sufficient to maintain normal adenylate levels in Burn-Vehicle presumably due to decreased availability of substrates, including PRPP and glutamine.

Uric acid is the end product of purine degradation (Figure S3). Uric acid was significantly higher in Burn-Vehicle compared with Sham-Vehicle and Burn-FTI (Figure 5). Among other purine degradative products, guanosine was significantly higher in Burn-Vehicle than Sham-Vehicle and Burn-FTI, while there were no significant differences in adenosine, inosine, and xanthine between Sham+Vehicle and Burn-Vehicle (Figure S4). Hypoxanthine tended to be lower in Burn-Vehicle compared with Sham-Vehicle, although there was no significant difference (*p* < 0.10) (Figure S4). Together, it is possible that the degradation of purines may be increased in addition to the decreased purine synthesis in Burn-Vehicle.

**Figure 6:**
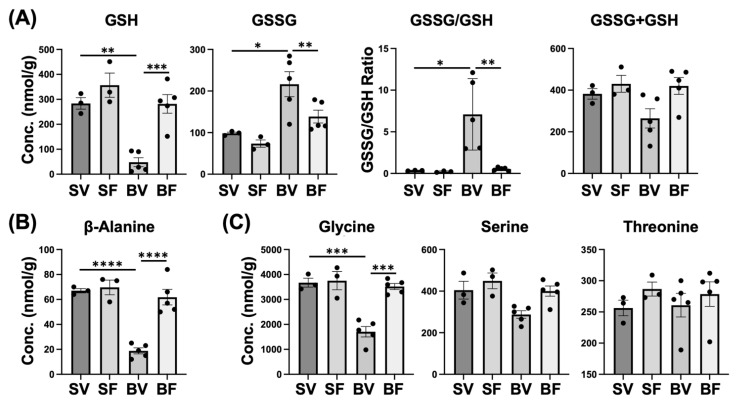
Effects of burn injury and FTI-277 on metabolites related to glutathione (**A**), β-alanine (**B**), glycine, serine, and threonine metabolism (**C**). SV: Sham-Vehicle; SF: Sham-FTI; BV: Burn-Vehicle; BF: Burn-FTI, GSH: reduced glutathione, GSSG: oxidized glutathione, * *p* < 0.05, ** *p* < 0.01, *** *p* < 0.001, **** *p* < 0.0001.

Related to glutathione metabolism, reduced glutathione (GSH) was markedly lower in Burn-Vehicle compared with Sham-Vehicle and Burn-FTI, while oxidized glutathione (GSSG) was significantly higher in Burn-Vehicle compared with Sham-Vehicle and Burn-FTI (Figure 6A). As a result, the GSSG/GSH ratio was markedly increased in Burn-Vehicle compared with Sham-Vehicle and Burn-FTI. The increases in GSSG and the GSSG/GSH ratio presumably reflect oxidative stress in Burn-Vehicle [7,29]. The sum of GSH and GSSG (GSH + GSSG) did not significantly differ between the four groups, although GSH + GSSG appeared to be lower in Burn-Vehicle (*p* > 0.10). Related to β-alanine metabolism, β-alanine and carnosine were markedly lower in Burn-Vehicle compared with Sham-Vehicle and Burn-FTI (Figure 6B and Figure S1). Related to glycine, serine, and threonine metabolism, glycine was significantly lower in Burn-Vehicle compared with Sham-Vehicle and Burn-FTI (Figure 6C). Serine tended to be lower in Burn-Vehicle compared with Sham-Vehicle, although there was no significant difference (*p* < 0.10). Threonine did not significantly differ between the four groups.

In all of these pathways, one or more metabolite (s) was significantly lower in Burn-Vehicle compared with Sham-Vehicle.

### 2.3. Other Metabolites and Pathways of Interest

**Figure 7:**
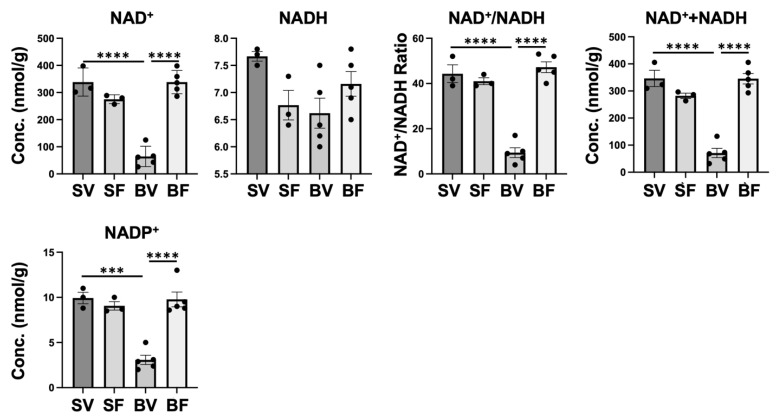
Effects of burn injury and FTI-277 on redox-related metabolites. SV: Sham-Vehicle; SF: Sham-FTI; BV: Burn-Vehicle; BF: Burn-FTI, *** *p* < 0.001, **** *p* < 0.0001.

In addition to the above-mentioned pathways, biologically interesting changes in additional metabolite levels were found, which include those reflecting redox status. NAD^+^ was markedly lower in Burn-Vehicle compared with Sham-Vehicle and Burn-FTI, while NADH did not significantly differ between the four groups (Figure 7). As a result, the NAD^+^/NADH ratio was markedly lower in Burn-Vehicle compared with Sham-Vehicle and Burn-FTI. The sum of NAD^+^ and NADH (NAD^+^ + NADH) was also markedly lower in Burn-Vehicle compared with Sham-Vehicle and Burn-FTI. Decreases in NAD^+^ and the NAD^+^/NADH ratio inhibit the activities of NAD^+^-dependent enzymes. We have previously shown [30] that burn injury increases acetylation of p53 and p65 nuclear factor (NF)-κΒ in mouse skeletal muscle, which, in turn, promotes apoptosis and inflammatory response, respectively. Both p53 and p65 NF-κΒ are deacetylated by Sirt1, an NAD^+^-dependent deacetylase [31]. Therefore, decreases in NAD^+^ and the NAD^+^/NADH ratio might contribute to increased acetylation of p53 and p65 NF-κΒ, and thereby apoptosis and inflammation in skeletal muscle in burned mice. Similarly, NADP^+^ was significantly lower in Burn-Vehicle compared with Sham-Vehicle and Burn-FTI. NADPH was undetectable in four of five mice in Burn-Vehicle and in one of five mice in Burn-FTI, while it was detectable in all of the mice in Sham-Vehicle and Sham-FTI. In our previous studies [23,26], burn injury induces mitochondrial dysfunction in skeletal muscle, which includes suppressed activity of complex I in the mitochondrial electron transport chain. A decrease in the NAD^+^/NADH ratio in Burn-Vehicle is consistent with decreased complex I activity, since complex I converts NADH to NAD^+^. In addition, mitochondrial dysfunction may contribute to a marked decrease in ATP levels in Burn-Vehicle (Figure 5). Our previous study [23] has shown that FTI-277 reverses burn injury-induced mitochondrial dysfunction, including complex I activity. Consistently, FTI-277 reversed the decreases in NAD^+^, the NAD^+^/NADH ratio, NAD^+^ + NADH, NADP^+^, and ATP to the levels in sham animals (Figure 5 and Figure 7).

**Figure 8:**
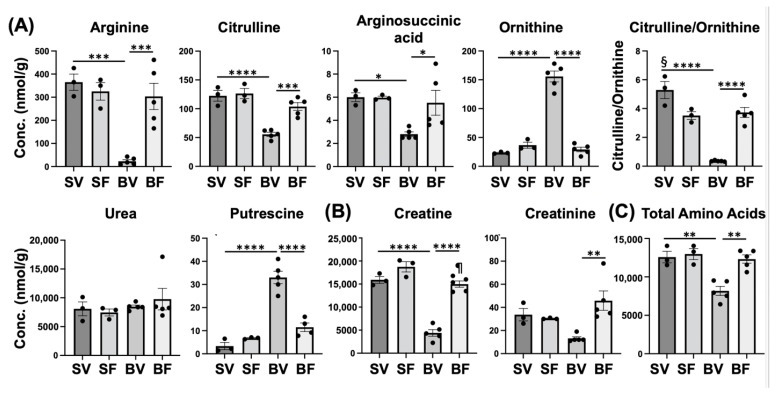
Effects of burn injury and FTI-277 on metabolites related to the urea/ornithine cycle (**A**), creatine metabolism (**B**), and total amino acids (**C**). SV: Sham-Vehicle; SF: Sham-FTI; BV: Burn-Vehicle; BF: Burn-FTI, * *p* < 0.05, ** *p* < 0.01, *** *p* < 0.001, **** *p* < 0.0001, § *p* < 0.05 vs. Sham-FTI and Burn-FTI, ¶ *p* < 0.05 vs. Sham-FTI.

Another example of altered metabolite levels is reflected in the urea/ornithine cycle, specifically arginine, citrulline, and ornithine. Arginine was markedly lower in Burn-Vehicle compared with Sham-Vehicle and Burn-FTI (Figure 8A). Nitric oxide synthase (NOS) converts arginine to nitric oxide and citrulline (Figure S5). Burn injury causes robust induction of iNOS in mouse skeletal muscle [19,30]. Hence, increased consumption of arginine by iNOS may be a contributing factor to decreased arginine in Burn-Vehicle. When arginine is metabolized by iNOS, it would increase citrulline. However, citrulline was significantly lower in Burn-Vehicle compared with Sham-Vehicle and Burn-FTI. In the urea/ornithine cycle (Figure S5), citrulline is converted to arginosuccinic acid. Arginosuccinic acid was also significantly lower in Burn-Vehicle compared with Sham-Vehicle and Burn-FTI. On the other hand, ornithine was markedly higher in Burn-Vehicle compared with Sham-Vehicle and Burn-FTI, while urea did not differ between the four groups. As a result, the citrulline/ornithine ratio was markedly lower in Burn-Vehicle compared with Sham-Vehicle and Burn-FTI. As ornithine transcarbamylase (OTC) converts ornithine to citrulline in the urea/ornithine cycle (Figure S5), it is possible that the conversion of ornithine to citrulline by OCT may be inhibited in Burn-Vehicle. Ornithine is also converted to putrescine by ornithine decarboxylase. Putrescine was markedly higher in Burn-Vehicle compared with Sham-Vehicle and Burn-FTI (Figure 8A).

Creatine was markedly lower in Burn-Vehicle compared with Sham-Vehicle and Burn-FTI (Figure 8B). Phosphocreatine and creatinine appeared to be lower in Burn-Vehicle compared with Sham-Vehicle, but there were no significant differences (*p* > 0.10) (Figure 8B and Figure S6). Creatine was considered to be mainly produced and released into the circulation by the liver and then taken up by skeletal muscle and other tissues [32]. However, the de novo synthesis of creatine occurs in skeletal muscle, as well, which is thought to contribute to the maintenance of the physiological level of creatine in this organ [33]. The first step of the de novo creatine synthesis is a conversion from glycine and arginine to ornithine and guanidinoacetic acid (GAA). GAA is then converted to creatine by methylation. Arginine and glycine were markedly lower in Burn-Vehicle (Figure 6C and Figure 8A), while ornithine was markedly higher in Burn-Vehicle (Figure 8A). It is conceivable that these changes in arginine, glycine, and ornithine levels may inhibit the first step of the de novo creatine synthesis.

Protein breakdown exceeds protein synthesis after burn injury, releasing amino acids from proteins [34]. Amino acids secreted into the circulation by the skeletal muscle are used as a substrate for gluconeogenesis in the liver. Our previous study [20] has shown that burn injury increases ex vivo amino acids released from skeletal muscle, which is reversed by FTI-277. Total amino acids were significantly lower in Burn-Vehicle compared with Sham-Vehicle and Burn-FTI (Figure 8C). Glycine (Figure 6C), arginine (Figure 8A), glutamine (Figure S1), alanine, histidine, and lysine were significantly lower in Burn-Vehicle compared with Sham-Vehicle, while methionine, phenylalanine, tryptophan, and tyrosine were significantly higher in Burn-Vehicle compared with Sham-Vehicle (Figure S7). It is conceivable that increased amino acid secretion from skeletal muscle may contribute to lower total amino acids in Burn-Vehicle.

### 2.4. The Warburg Effect and Glycolysis 

**Figure 9:**
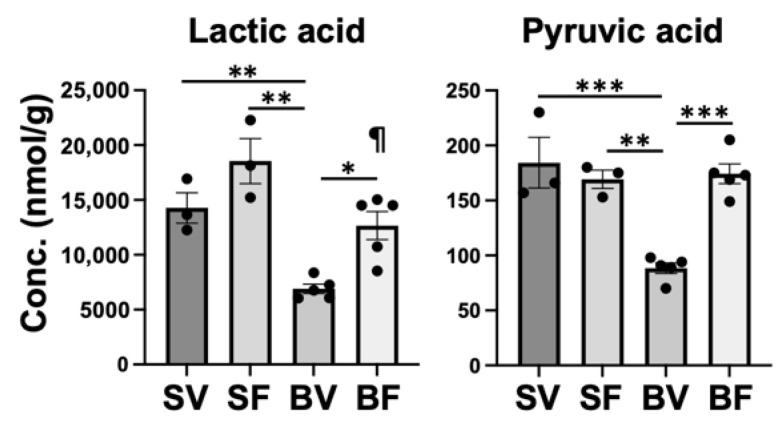
Effects of burn injury and FTI-277 on lactic acid and pyruvic acid. SV: Sham-Vehicle; SF: Sham-FTI; BV: Burn-Vehicle; BF: Burn-FTI, * *p* < 0.05, ** *p* < 0.01, *** *p* < 0.001, ¶ *p* < 0.05 vs. Sham + FTI.

Our previous studies have shown [20,23] that burn injury induces the Warburg effect in mouse skeletal muscle, as indicated by robust increases in expression of HIF-1α and its downstream genes. The burn injury-induced Warburg effect is reversed by FTI-277 [20,23]. The Warburg effect increases lactate synthesis and secretion. Consistently, burn injury markedly increases ex vivo secretion of lactate by skeletal muscle and circulating lactate levels in mice, which are associated with increased mRNA levels of LDHA [23]. Unexpectedly, however, lactic acid was significantly lower in Burn-Vehicle compared with Sham-Vehicle and Burn-FTI (Figure 9). Monocarboxylate transporter 4 (MCT4), a downstream gene of HIF-1α [35], plays an important role in lactic acid secretion [36]. It is possible that the lower lactic acid in Burn-Vehicle could be attributable at least in part to increased MCT-mediated lactic acid secretion that exceeds lactate synthesis. Pyruvic acid was significantly lower in Burn-Vehicle compared with Sham-Vehicle and Burn-FTI. Pyruvic acid is converted to acetyl-CoA and to lactic acid by PDH and LDH, respectively. The Warburg effect is associated with decreased PDH activity and increased LDH activity [37]. Therefore, it is possible that decreased lactic acid levels may contribute to lower pyruvic acid levels in Burn-Vehicle.

Among glycolysis intermediates, glucose-6-phosphate (G6P) and fructose-6-phosphate (F6P) did not significantly differ between Sham-Vehicle and Burn-Vehicle, while G6P and F6P were significantly higher in Burn-FTI compared with the other three groups (Figure S8). On the other hand, fructose-1,6-bisphosohate (F1,6P) and 3-phosphoglyceric acid (3-PG) did not significantly differ between the four groups, although they appeared to be higher in Sham-Vehicle and Burn-FTI compared with Sham-FTI and Burn-Vehicle (*p* > 0.10). Glycerol 3-phosphate (G3P) was significantly lower in Burn-Vehicle compared with Sham-Vehicle and Burn-FTI (Figure S8).

### 2.5. Glutaminolysis and Reductive Carboxylation 

**Figure 10:**
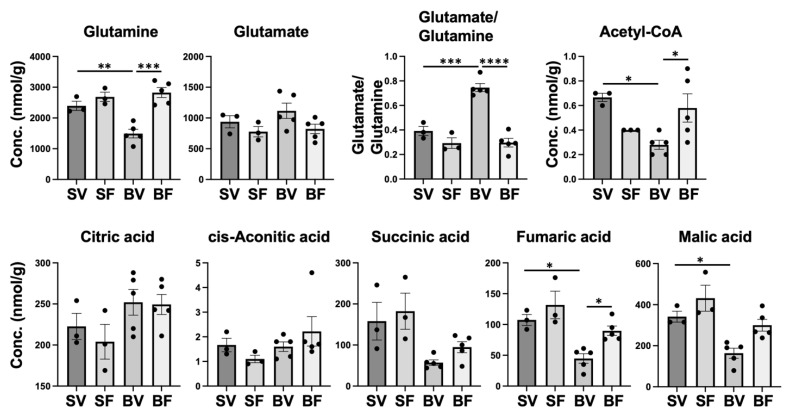
Effects of burn injury and FTI-277 on glutamine, glutamate, and TCA cycle intermediates. SV: Sham-Vehicle; SF: Sham-FTI; BV: Burn-Vehicle; BF: Burn-FTI, * *p* < 0.05, ** *p* < 0.01, *** *p* < 0.001, **** *p* < 0.0001.

The Warburg effect is often accompanied by increased glutaminolysis [38,39,40] and reductive carboxylation of glutamine to form citric acid [41] (Figure S9). Previous studies [42,43,44] have shown that circulating glutamine concentrations are lower in burn patients and burned animals compared with respective healthy controls. Skeletal muscle is the main glutamine-producing tissue, accounting for 90% of all glutamine synthesis [45]. Glutamine levels in skeletal muscle are decreased in critically ill patients, although glutamine synthesis in skeletal muscle is increased and glutamine secretion into the circulation is similar to that of healthy controls [46]. These results suggest that glutamine consumption may be increased in skeletal muscle in critically ill patients. Similarly, glutamine levels in skeletal muscle are decreased after burn injury in rats [47]. Clinical trials of glutamine supplementation showed benefits in burn patients, including significant reductions of gram-negative bacteremia [48] and amelioration of the hypermetabolic response and organ damage [49]. These results indicate that the altered glutamine metabolism has a real impact on burn patients. Consistently, glutamine was significantly lower in Burn-Vehicle compared with Sham-Vehicle and Burn-FTI (Figure 10). It remains to be clarified how to burn injury increases glutamine consumption in skeletal muscle. Glutaminolysis is the process by which cells convert glutamine into tricarboxylic acid (TCA) cycle intermediates via α-ketoglutarate through the activities of multiple enzymes (Figure S9). The first step of glutaminolysis is the conversion of glutamine to glutamate by glutaminase. In this study, glutamate did not significantly differ between the four groups (*p* > 0.10), although the average of glutamate levels appeared to be higher in Burn-Vehicle compared with Burn-FTI. As a result, the glutamate/glutamine ratio was significantly higher in Burn-Vehicle compared with Sham-Vehicle and Burn-FTI. The increased glutamate/glutamine ratio is consistent with increased glutaminolysis [50]. A previous study has shown in rats [43] that burn injury decreases plasma glutamine levels and that glutamine supplementation increases plasma levels of glutamate and α-ketoglutarate as well as glutamine in burned rats. These data indicate that glutamine can be efficiently converted to glutamate and α-ketoglutarate in burned rodents. Together, our data suggest that increased glutaminolysis may contribute to low levels of glutamine in skeletal muscle and in the circulation in burn patients and burned rodents. However, our data cannot exclude the possibility that increased secretion of glutamine into the circulation may play a role in low glutamine levels in skeletal muscle in Burn-Vehicle.

Glutaminolysis activates mTORC1 [51,52], while mTORC1 activation promotes glutaminolysis [53]. In our previous study [23], mTORC1 is markedly activated in mouse skeletal muscle after burn injury, which is reversed by FTI-277. mTORC1 activation induces insulin resistance [54] and HIF-1α expression [55], the latter of which leads to the Warburg effect. Thus, it is conceivable that increases in glutaminolysis and mTORC1 activity form a vicious cycle, which is considered to contribute to insulin resistance and the Warburg effect in mouse skeletal muscle after burn injury.

Glutamate is converted to α-ketoglutarate, which replenishes TCA cycle intermediates (anaplerosis) and provides carbon for reductive carboxylation (Figure S9). When the Warburg effect is operative, conversion of pyruvate to acetyl CoA by PDH is inhibited by PDK-mediated phosphorylation of PDH. As mentioned above, burn injury increases PDK1 expression in mouse skeletal muscle [23]. In line with PDH inhibition, acetyl-CoA was significantly lower in Burn-Vehicle compared with Sham-Vehicle and Burn-FTI (Figure 10). Among TCA cycle intermediates, citric acid, cis-aconitic acid, succinic acid, fumaric acid, and malic acid were detected. α-ketoglutarate was not detectable in all the groups. In contrast to the lower acetyl-CoA levels in Burn-Vehicle, there were no differences in citric acid and cis-aconitic acid between the four groups. On the other hand, fumaric acid and malic acid were significantly lower in Burn-Vehicle compared with Sham-Vehicle. Succinic acid tended to be lower in Burn-Vehicle compared with Sham-Vehicle, although there was no significant difference (*p* < 0.10). This pattern of the concentrations of TCA cycle intermediates is consistent with increased reductive carboxylation of α-ketoglutarate to generate citric acid [56,57]. The suppressed NAD^+^/NADH ratio (Figure 7) is considered to promote reductive carboxylation of α-ketoglutarate since NADH is converted to NAD^+^ during reductive carboxylation [58,59].

Glutaminolysis and reductive carboxylation have been shown to support cancer cell growth [38,60,61] and insulin secretion from pancreatic β cells [62]. In macrophages, lipopolysaccharide (LPS) increases glutaminolysis [63]. While glutaminolysis restricts M1 polarization, α-ketoglutarate produced from glutaminolysis during LPS stimulation has a crucial role in promoting LPS-induced endotoxin tolerance in macrophages [56,63,64]. However, the role of glutaminolysis and reductive carboxylation in skeletal muscle remains largely unknown. Previous studies have shown that accelerated glutaminolysis and reductive carboxylation are adaptive in a mouse model of mitochondrial myopathy [65] and in cells with mitochondrial DNA mutation [66] and mitochondrial damage under hemorrhagic shock [67]. Together, it is tempting to speculate that increased glutaminolysis and reductive carboxylation may be an adaptive response to mitochondrial damage after burn injury, partly because NADH is converted to NAD^+^ during reductive carboxylation ameliorating redox imbalance. Of note, a previous study [68] has shown that administration of dichloroacetate, an inhibitor of PDK, increases glutamine levels in skeletal muscle while decreasing circulating lactate levels in burn patients. Together, these results support the notion that the Warburg effect and resultant decreased PDH activity may contribute to low glutamine levels in skeletal muscle after burn injury presumably by increasing glutaminolysis.

In the present study, the metabolomics data analysis indicates that burn injury induced a global change in metabolome composition and metabolic pathways and that one of the characteristics of burn injury-induced metabolic alterations is substantial decreases in abundance of some key metabolites in multiple metabolic pathways, although some other metabolites were significantly increased in Burn-Vehicle compared with Sham-Vehicle.

### 2.6. Effects of FTI-277

Overall, our data showed that FTI-277 almost completely reversed burn injury-induced changes in metabolite levels. These results indicate that the beneficial effects of FTI-277 in burned mice are not limited to the reversal of insulin resistance, the Warburg effect, and mitochondrial dysfunction as shown in our previous studies [20,23]. Rather, FTI-277 reversed the burn injury-induced global changes in metabolism in mouse skeletal muscle.

In contrast to the overt effects of FTI-277 in burned mice, those of FTI-277 are limited in sham-burned mice. As stated above, between Sham-Vehicle and Sham-FTI there were no differential metabolites, which were defined as those with an absolute fold change > 2 and *p*-value < 0.05. However, FTI-277 significantly increased levels of IMP and succinyl AMP in sham animals (Figure 5) though the fold changes were less than two.

### 2.7. Limitations of This Study

This study has some limitations. First, we performed metabolomics analysis at one-time point after burn injury. We examined the metabolome at 3 days after burn injury because the maximum effects of burn injury on insulin resistance and HIF-1α expression in skeletal muscle and on hyperlactatemia were observed at 3 days after burn injury [19,20,23]. However, this study cannot tell time-dependent changes in metabolite levels after burn injury. Second, the numbers of animals used in this study are small, although they were sufficient to find statistical differences in many metabolites between the groups. Third, we did not evaluate the flux of metabolites using stable isotope-labeled substrates. The metabolomics analysis is illustrative of a snapshot of levels of metabolites at one point in time. However, many reactions between metabolites are bidirectional, which include conversions between TCA cycle intermediates and between glutamine and glutamate. Therefore, flux studies using stable isotope-labeled glutamine are required to confirm that burn injury induces increased glutaminolysis and reductive carboxylation in skeletal muscle. All results and discussions should be interpreted in light of these limitations.

## 3. Materials and Methods

### 3.1. Animals

All experiments were carried out in accordance with the institutional guidelines and the study protocol was approved by the Institutional Animal Care and Use Committee (IACUC) at the Massachusetts General Hospital (the protocol title: Stress-Associated Insulin Resistance; the protocol#: 2007N000020). The animal care facility is accredited by the Association for Assessment and Accreditation of Laboratory Animal Care.

We used male C57BL/6 mice (Jackson Laboratory, Bar Harbor, ME, USA) at 8 weeks of age. The mice were housed in a pathogen-free animal facility with 12 h light/dark cycles at 22 °C. A full-thickness burn injury comprising 30% of total body surface area was produced under anesthesia with pentobarbital sodium (50 mg/kg BW, IP) in mice by immersing the abdomen for 6 s and both sides of the flank for 4 s in 80 °C water as previously described [19,20,23]. We confirmed that this procedure produced full-thickness third-degree burn injury in mice by hematoxylin and eosin staining. Sham-burned mice were immersed in lukewarm water. Buprenorphine (0.1 mg/kg BW, SC) was administered every 8–12 h up to 72 h after burn or sham-burn. For resuscitation, prewarmed normal saline (0.04 mL/g BW, IP) was injected just after burn injury or sham-burn regardless of the treatments. Starting at 2 h after burn injury or sham-burn, the mice were treated with FTI-277 (N-[4-[2(R)-amino-3-mercaptopropyl]amino-2-phenylbenzoyl] methionine methyl ester trifluoroacetate salt) (5 mg/kg BW/day, IP, Sigma, St. Louis, MO, USA) or vehicle (phosphate-buffered saline [PBS]) for 3 days. The numbers of animals in the four groups were: (1) sham-burned mice treated with vehicle: *n* = 3; (2) sham-burned mice treated with FTI-277: *n* = 3; (3) burned mice treated with vehicle: *n* = 5; and (4) burned mice treated with FTI-277: *n* = 5. None of the mice died or became moribund after burn injury. At 3 days after burn injury or sham-burn, rectus abdominis muscle was excised under anesthesia, snap-frozen and kept at −80 °C freezer until analyzed. Approximately 50 mg of muscle samples were used for metabolomics analysis.

### 3.2. Metabolomics Procedure

A total of 116 metabolites (54 and 62 metabolites in the cation and anion modes, respectively) involved in glycolysis, pentose phosphate pathway, TCA cycle, urea cycle, and polyamine, creatine, purine, glutathione, nicotinamide, choline, and amino acid metabolism were analyzed. Among the 116 metabolites, 102 metabolites were detected in mouse skeletal muscle samples (Table S2).

The metabolomics procedure was performed at Human Metabolome Technologies (HMT) (Tsuruoka, Yamagata, Japan) as previously described [69,70,71] with minor modifications. Briefly, the muscle samples were mixed with 1500 μL of 50% acetonitrile in water (*v*/*v*) containing internal standards (20 μM for cation and 5 μM for anion measurement), homogenized by a homogenizer (MS-100R, TOMY Digital Biology, Tokyo, Japan) with beads, and centrifuged (4000 rpm, 60 s × 6 times). The supernatant (400 μL × 2) was then filtrated through 5-kDa cut-off filter (ULTRAFREE-MC-PLHCC, Human Metabolome Technologies) to remove macromolecules. The filtrate was centrifugally concentrated and resuspended in 50 μL of ultrapure water immediately before the measurement.

Targeted quantitative analysis was performed using capillary electrophoresis mass spectrometry (CE-MS) in mouse skeletal muscle samples. Cationic metabolites were measured in the cation mode of metabolome analysis using the Agilent CE-TOFMS system (Agilent Technologies, Santa Clara, CA, USA), while anionic metabolites were measured in the anion mode of metabolome analysis using Agilent 6460 TripleQuad LC/MS (Agilent Technologies). A total of 116 metabolites (Table S2) were analyzed using a fused silica capillary (50 μm i.d. × 80 cm total length) with commercial electrophoresis buffer (Solution ID: H3301-1001 for the cation analysis and H3302-1021 for the anion analysis, Human Metabolome Technologies) as the electrolyte. The samples were diluted 2-fold and 10-fold to improve analysis qualities in the cation and the anion modes of the CE-MS analysis, respectively. Then, the sample was injected at a pressure of 50 mbar for 10 s for the cation analysis and 25 s for the anion analysis. Peaks detected in CE-TOFMS analysis were extracted using an automatic integration software (MasterHands ver.2.16.0.15 developed at Keio University, Tokyo, Japan) [72] and those in CE-MS/MS analysis were extracted using an automatic integration software (MassHunter Quantitative Analysis B.06.00, Agilent Technologies) in order to obtain peak information including *m/z*, migration time (MT) and peak area. Putative metabolites were then assigned from the HMT metabolite database on the basis of *m/z* and MT. The tolerance was ±0.5 min in MT and ±10 ppm in *m/z*. All the metabolite concentrations were calculated by normalizing the peak area of each metabolite with respect to the area of the internal standard and by using standard curves, which were obtained by three-point calibrations. Concentrations of metabolites were normalized to the mass of the muscle samples and are expressed as nmol/g tissue.

### 3.3. Metabolomics Data Analysis

Principal component analysis (PCA) was performed using *prcomp* from the stats R package. Partial least squares discriminant analysis (PLS-DA) was conducted using the *PLSDA.CV* function in R package MetaboAnalystR. *hclut* function of stats R package was adopted for hierarchical clustering in the heatmap. For differential metabolite analysis, the Wilcoxon rank sum test and Student *t*-test were performed using the wilcox R function and *t*-test R function, respectively, to infer differential metabolites between two compared groups. R package MetaboAnalystR was used for pathway enrichment analysis based on the murine KEGG pathway reference database. A hypergeometry test was chosen as the enrichment method. Pathway impact was calculated as the sum of the important measures of the matched metabolites normalized by the sum of the important measures of all metabolites in each pathway.

### 3.4. Statistical Analysis

Differential metabolites between two groups were determined by the Wilcoxon rank sum test and Student’s *t*-test using wilcox R function and *t*-test R function of stats R package, respectively. Differential metabolites were defined as those with an absolute fold change > 2 and *p*-value < 0.05. Fold change is the ratio of the mean of metabolites of the first group to that of the second group. The four groups were compared by one-way ANOVA followed by Tukey’s multiple comparison test using Prism 9 (GraphPad Software, San Diego, CA, USA). Data are expressed as mean ± SEM. *p* < 0.05 was considered statistically significant.

## 4. Conclusions

Metabolomics analysis showed that skeletal muscle underwent substantial metabolic alterations after burn injury, as compared with sham animals. FTI-277 treatment almost completely reversed the burn-induced changes in metabolite levels, while it did not alter metabolome profile in sham animals. Overall, this study suggests that burn injury-induced alterations of metabolic pathways may be characterized by a deficiency of some metabolites that are important for respective metabolic pathways, although some other metabolites were increased after burn injury. Moreover, our data raise the possibility that burn injury may lead to increased glutaminolysis and reductive carboxylation, which is often associated with the Warburg effect. However, further studies are required to clarify this point.

## Figures and Tables

**Table 1:** The top ten metabolites with the highest absolute factor loadings on the first principal component (PC1).

Metabolite	PC1 Loading
Creatine	−0.768436391
Lactic acid	−0.589380214
ATP	−0.182583912
Glycine	−0.120121284
Glutamine	−0.06894234
Inosine 5′-monophosphate	−0.048388868
Alanine	−0.0446261
Carnosine	−0.03553214
Lysine	−0.033458763
Glycerol-3-phosphate	−0.026523959

**Table 2:** Metabolic pathways with significant changes.

Pathway	Raw p	FDR	Impact
Purine metabolism	0.0000000	0.0000000	0.32877
Glutathione metabolism	0.0000176	0.0007371	0.15168
β-Alanine metabolism	0.0000326	0.0009118	0.56716
Glycine, serine and threonine metabolism	0.0005840	0.0122640	0.47182
Aminoacyl-tRNA biosynthesis	0.0037617	0.0631960	0.00000
Pentose phosphate pathway	0.0047003	0.0658050	0.30436
Phenylalanine, tyrosine and tryptophan biosynthesis	0.0059698	0.0694750	1.00000
Arginine and proline metabolism	0.0066167	0.0694750	0.04613
Arginine biosynthesis	0.0091558	0.0854540	0.34518
Alanine, aspartate and glutamate metabolism	0.0113930	0.0957030	0.04566
Pantothenate and CoA biosynthesis	0.0217380	0.1660000	0.19643
TCA cycle	0.0249980	0.1749900	0.11283
Pyruvate metabolism	0.0322670	0.2084900	0.36081
Glycolysis / Gluconeogenesis	0.0497550	0.2985300	0.18635

## Data Availability

The data presented in this study are available in the article and Supplementary Materials.

## Supplementary Materials

The following supporting information can be downloaded at: https://www.mdpi.com/article/10.3390/metabo12090800/s1, Table S1: The top five metabolites with the highest positive factor loadings on the first principal component. Table S2: The list of 116 metabolites that were evaluated in this study. Figure S1: Effects of burn injury and FTI-277 on the top 10 metabolites with the highest absolute PC1 factor loadings. Figure S2: Intersection of differential metabolites. Figure S3: Purine metabolism. Figure S4: Effects of burn injury and FTI-277 on metabolites related to purine metabolism. Figure S5: Urea/Ornithine cycle. Figure S6: Effects of burn injury and FTI-277 on phosphocreatine levels. Figure S7: Effects of burn injury and FTI-277 on amino acids levels. Figure S8: Effects of burn injury and FTI-277 on glycolysis intermediates. Figure S9: TCA cycle.
