# Supplementary material for: Farnesysltransferase Inhibitor Prevents Burn Injury-Induced Metabolome Changes in Muscle
Source: Metabolites. 2022 Aug 27;12(9):800. doi: 10.3390/metabo12090800 (PMC9506277; doi:10.3390/metabo12090800)
Supplement: Supplementary file 1 [file metabolites-12-00800-s001.zip › metabolites-1820426-supplementary.pdf]

Table S1. The top five metabolites with the highest positive factor loadings on the first principal component (PC1).

Metabolite	PC1 loading
Glutamate	0.01683966
Ornithine	0.007082639
Oxidized glutathione	0.006829778
Aspartic acid	0.002970479
Leucine	0.002071246

Table S2. The list of 116 metabolites that were evaluated in this study. The asterisks indicate metabolites that were not detected in any samples.

NAD^+	6-Phosphogluconic acid	*N,N*-Dimethylglycine
cAMP	*N*-Carbamoylaspartic acid	γ-Aminobutyric acid
cGMP*	PRPP	Choline
NADH	2-Phosphoglyceric acid	Serine
Xanthine	2,3-Diphosphoglyceric acid	Creatinine
ADP-ribose	3-Phosphoglyceric acid	Proline
Mevalonic acid	Phosphoenolpyruvic acid	Valine
UDP-glucose	GMP	Betaine
Uric acid	AMP	Threonine
$NADP^+$	2-Oxoisovaleric acid	Homoserine
IMP	GDP	Betaine aldehyde*
Sedoheptulose 7-phosphate*	Lactic acid	Cysteine
Glucose 6-phosphate	ADP	Hydroxyproline
Fructose 6-phosphate	GTP	Creatine
Fructose 1-phosphate*	Glyoxylate*	Isoleucine
Galactose 1-phosphate	ATP	Leucine
Glucose 1-phosphate	Glycerol 3-phosphate	Asparagine
Acetoacetyl CoA*	Glycolic acid	Ornithine
Acetyl CoA	Pyruvic acid	Aspartic acid
Folic acid*	*N*-Acetylglutamic acid	Homocysteine*
Ribose 5-phosphate	2-Hydroxyglutaric acid	Adenine
CoA	Carbamoylphosphate*	Hypoxanthine
Ribose 1-phosphate	Succinic acid	Spermidine
Ribulose 5-phosphate	Malic acid	Glutamine
Xylulose 5-phosphate	2-Oxoglutaric acid*	Lysine
Erythrose 4-phosphate*	Fumaric acid	Glutamate
HMG CoA	Citric acid	Methionine
Glyceraldehyde 3-phosphate	*cis*-Aconitic acid	Guanine*
NADPH	Isocitric acid*	Histidine
Malonyl CoA	Urea	Carnitine
Phosphocreatine	Glycine	Phenylalanine
XMP	Putrescine	Arginine
Dihydroxyacetone phosphate	β-Alanine	Citrulline
Adenylosuccinic acid	Alanine	Tyrosine
Fructose 1,6-diphosphate	Sarcosine	Spermine
Triptophan	Inosine	Glutathione (GSH)
Cystathionine	Guanosine	*S*-Adenosylhomocysteine*
Carnosine	Argininosuccinic acid	*S*-Adenosylmethionine
Adenosine	Glutathione (GSSG)	

The asterisks indicate metabolites that were not detected in any samples.

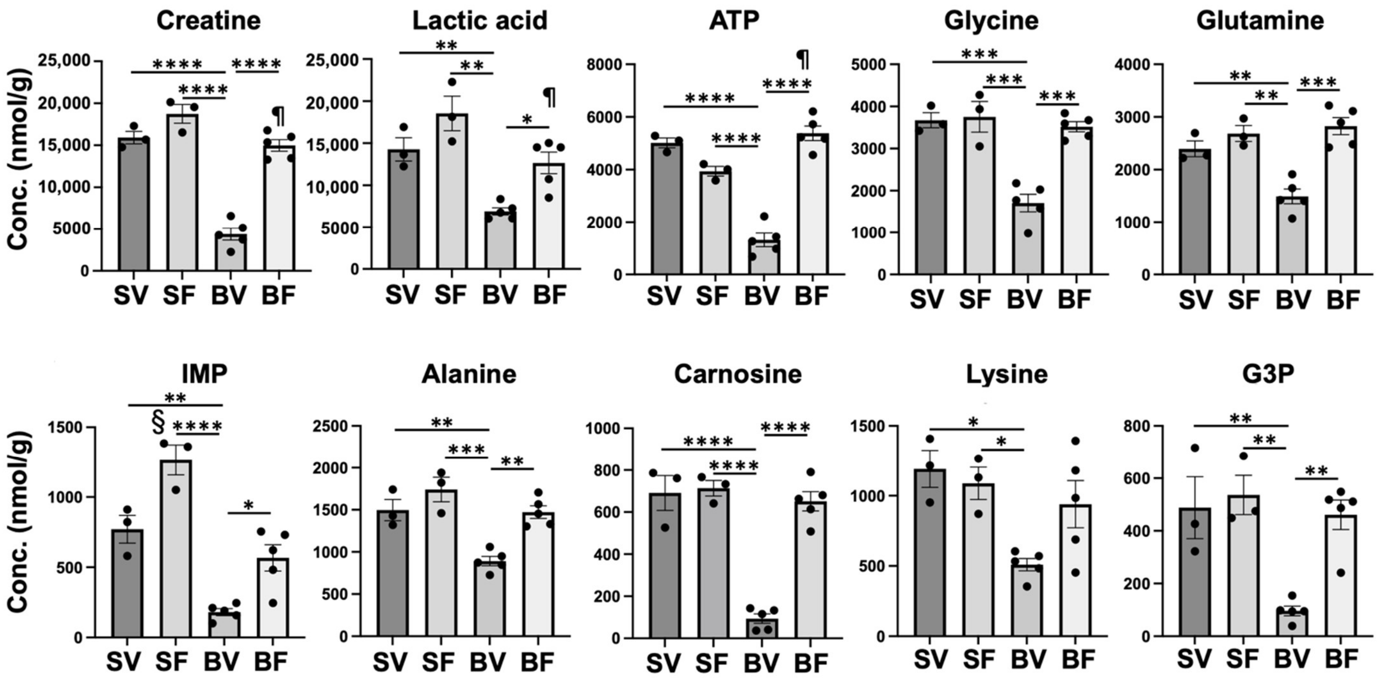

Figure S1. Effects of burn injury and FTI-277 on the top 10 metabolites with the highest absolute PC1 factor loadings. SV: Sham-Vehicle; SF: Sham-FTI; BV: Burn-Vehicle; BF: Burn-FTI, IMP: inosine 5′-monophosphate, G3P: glycerol-3-phosphate, *p<0.05, **p<0.01, ***p<0.001, ****p<0.0001, §p<0.05 vs. Sham-Vehicle, p<0.001 vs. Burn-FTI, ¶p<0.05 vs. Sham-FTI.

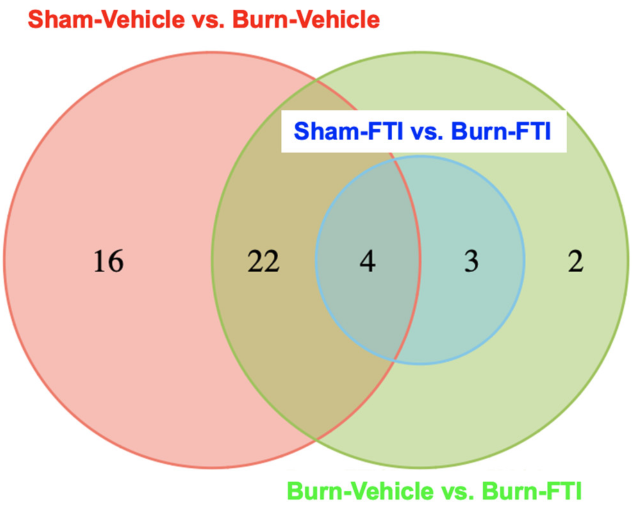

Figure S2. Intersection of differential metabolites. Venn diagram illustrates the distribution of metabolites showing significantly (p<0.05 by Student t test) modulated levels. Red, blue and green circles indicate differential metabolites between Sham-Vehicle vs. Burn-Vehicle, those between Sham-FTI vs. Burn-FTI and those between Burn-Vehicle vs. Burn-FTI, respectively.

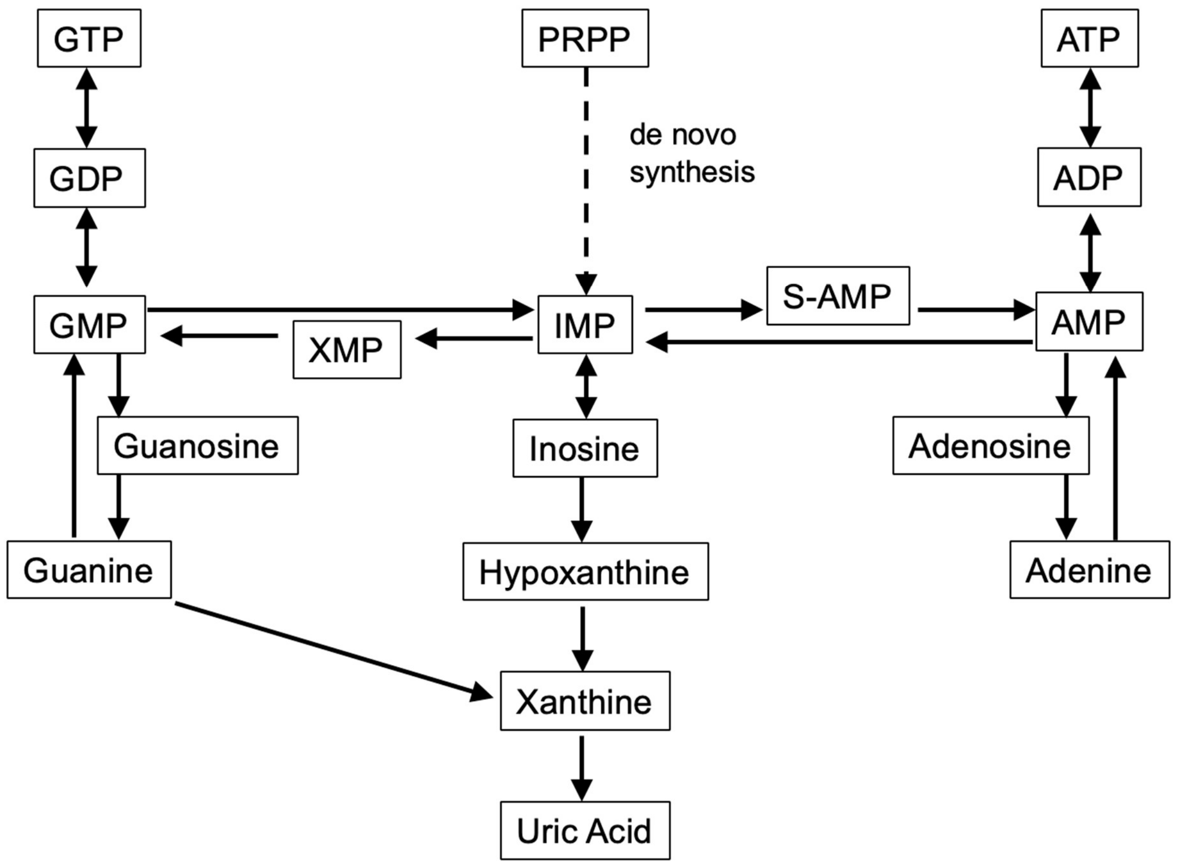

Figure S3. Purine metabolism. IMP: inosine 5′-monophosphate, PRPP: phosphoribosylpyrophosphate, S-AMP: succinyl AMP (adenylosuccinate), XMP: xanthine monophosphate.

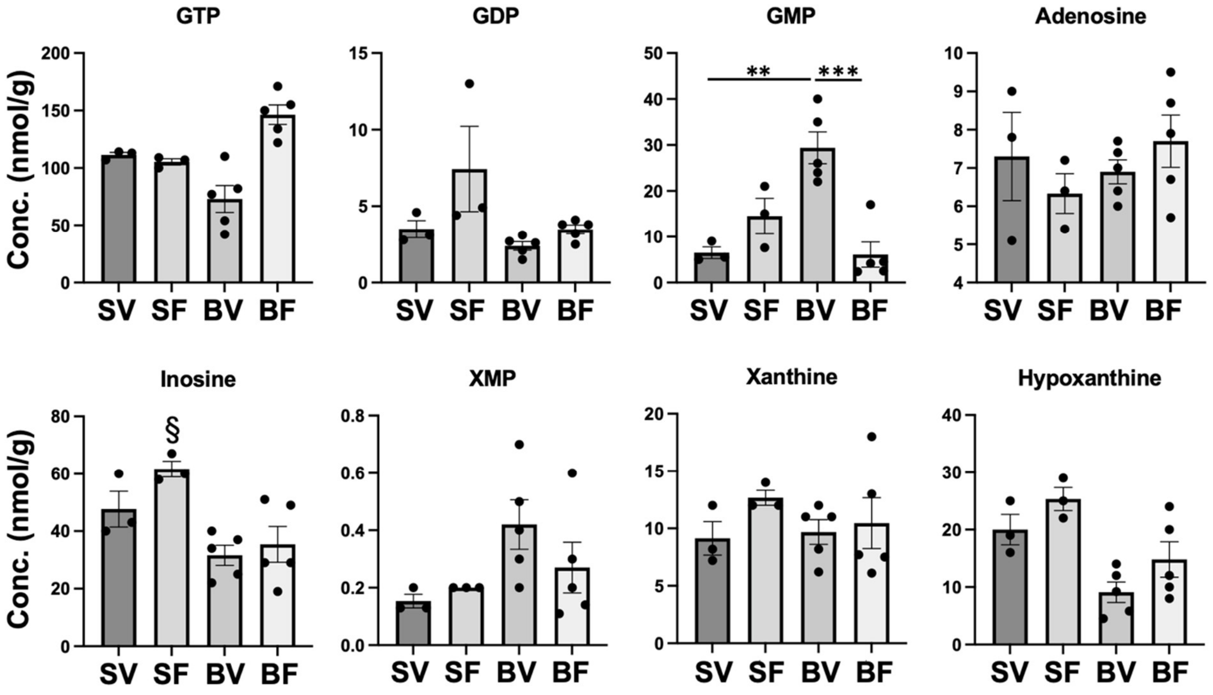

Figure S4. Effects of burn injury and FTI-277 on metabolites related to purine metabolism. SV: Sham-Vehicle; SF: Sham-FTI; BV: Burn-Vehicle; BF: Burn-FTI, XMP: xanthine monophosphate, **p<0.01, ***p<0.001, §p<0.05 vs. Burn-FTI.

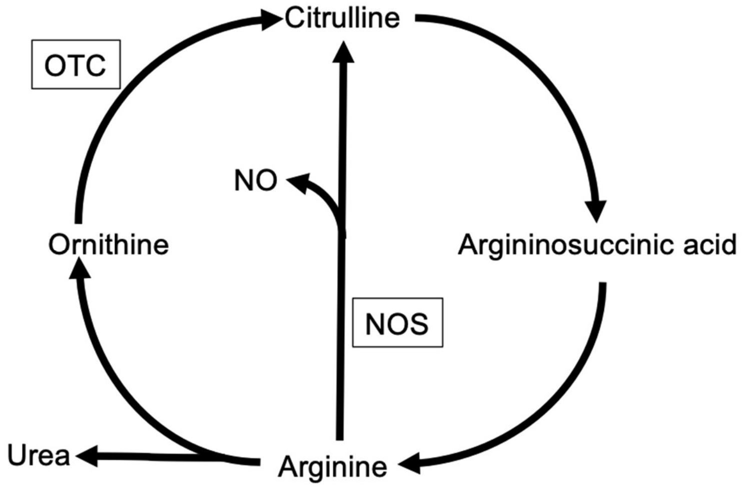

Figure S5. Urea/Ornithine cycle. NO: nitric oxide, NOS: nitric oxide synthase, OTC: ornithine transcarbamylase.

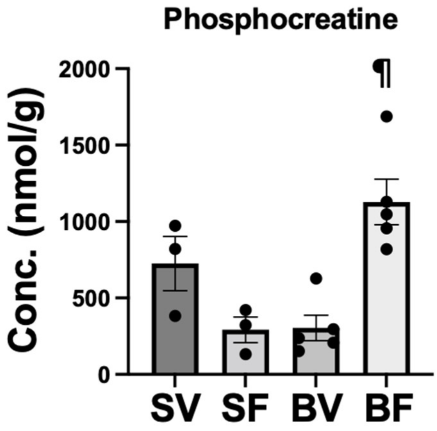

Figure S6. Effects of burn injury and FTI-277 on phosphocreatine levels. SV: Sham-Vehicle; SF: Sham-FTI; BV: Burn-Vehicle; BF: Burn-FTI, ¶p<0.01 vs. Sham-FTI & Burn-Vehicle.

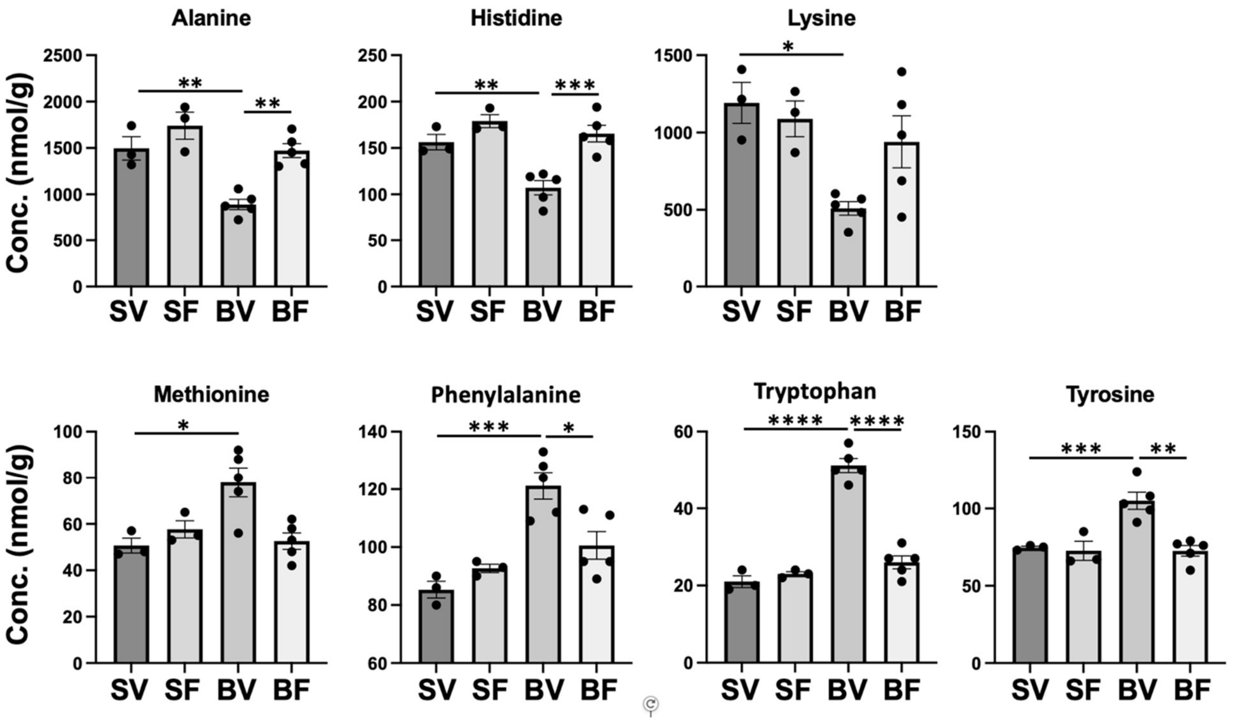

Figure S7. Effects of burn injury and FTI-277 on amino acids levels. SV: Sham-Vehicle; SF: Sham-FTI; BV: Burn-Vehicle; BF: Burn-FTI, *p<0.01, **p<0.01, ***p<0.001, ****p<0.0001.

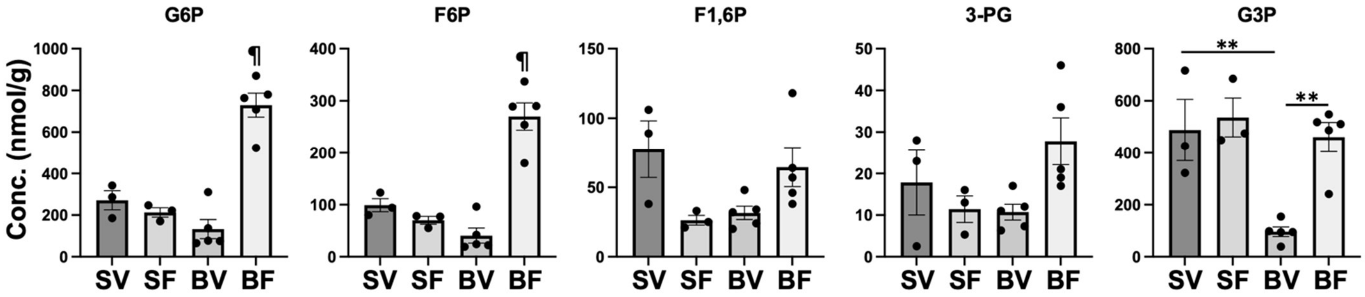

Figure S8. Effects of burn injury and FTI-277 on glycolysis intermediates. G6P: glucose-6-phosohate, F6P: fructose-6-phosohate, F1,6P: fructose-1,6-bisphosphate, 3-PG: 3-phosphoglyceric acid, G3P: glycerol 3-phosphate, **p<0.01, ¶p<0.001 vs. Sham-Vehicle, p<0.0001 vs. Sham-FTI & Burn-Vehicle.

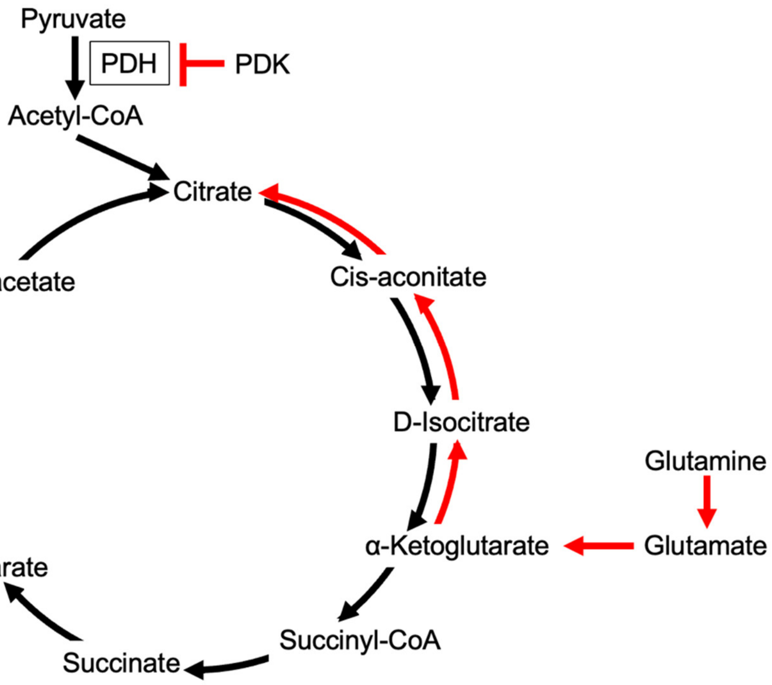

Figure S9. TCA cycle. Black arrows indicate reactions under a normal condition. Red arrows indicate glutaminolysis and reductive carboxylation. PDH: pyruvate dehydrogenase, PDK: pyruvate dehydrogenase kinase.
